# Vanishing spleen syndrome post right partial nephrectomy in a sicklemic patient

**DOI:** 10.11604/pamj.2018.30.211.15454

**Published:** 2018-07-16

**Authors:** Khuram Khan, Ofelia Leroux, Saqib Saeed, Farhana Iqbal, Leaque Ahmed, Brian Davis-Joseph

**Affiliations:** 1 Department of Surgery, Harlem Hospital Center, Columbia University, New York, NY, USA; 2 Department of Internal Medicine, Richmond University Medical Center, Staten Island, NY, USA; 3 Division of Urology, Harlem Hospital Center, Columbia University, New York, NY, USA

**Keywords:** Splenic infarction, right partial nephrectomy, laparoscopic renal surgery, sickle cell disease

## Abstract

Splenic infarction after contralateral laparoscopic renal surgery has not, to our knowledge, been reported. The spleen is the most affected organ in sickle cell disease and the mechanism of auto infarction is thought to result from the crystallization of abnormal hemoglobin during periods of hypoxia or acidosis resulting in parenchymal ischemia and ultimately tissue necrosis. We report a case of 45 year old female with sickle cell disease who had an unremarkable spleen at the time of a laparoscopic right partial nephrectomy and was subsequently found to have marked diminution in her splenic volume.

## Introduction

Spleen injury is an underestimate diagnosis of abdominal surgery. It is estimated that between 4 to 13% of splenic injury are iatrogenic. Splenic infarction after right partial nephrectomy is rare.

## Patient and observation

**Figure 1:**
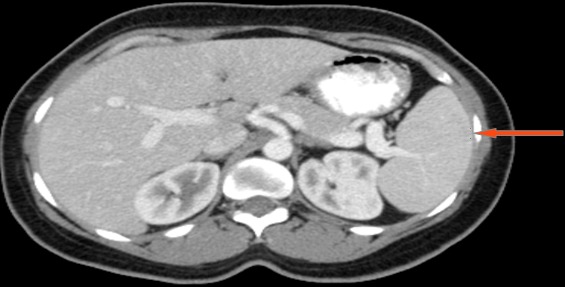
Pre-surgery axial abdomen CT: shows spleen (red arrow)

**Figure 2:**
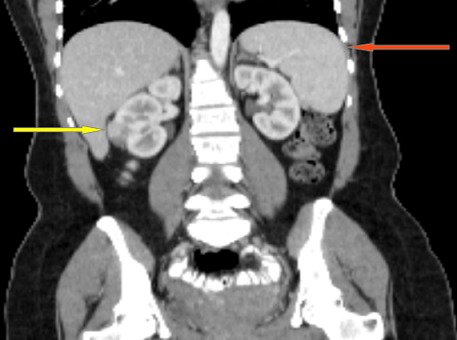
Pre-surgery coronal abdomen CT: shows spleen (red arrow), right renal mass (yellow arrow)

**Figure 3:**
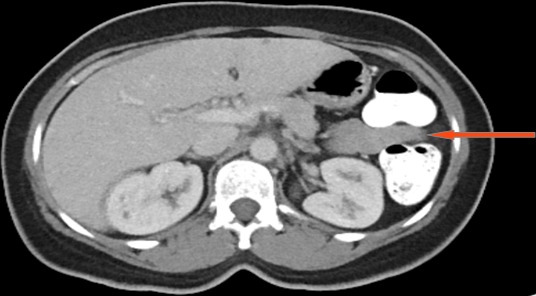
Post-surgery axial abdomen CT: shows several splenules, no spleen (red arrow)

**Figure 4:**
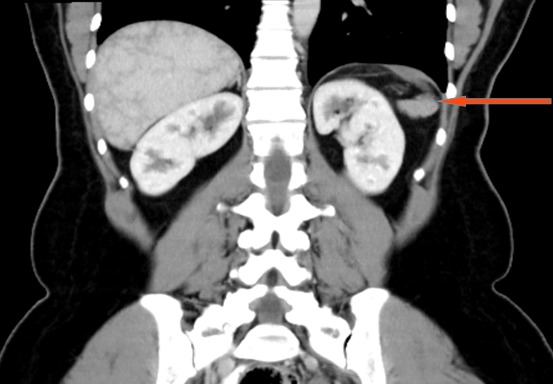
Post-surgery coronal abdomen CT: shows several splenules, no spleen (red arrow)

A 45 year old female with history of sickle cell disease presented with abdominal pain. A CT scan revealed an exophytic renal mass measuring 2.9cm x 2.0cm x 2.1cm located on the antero-lateral aspect of her right kidney and an unremarkable spleen (Figure 1, Figure 2). She was evaluated by urology and a right partial nephrectomy was planned, however on the day of the procedure, the patient's preoperative pregnancy test was positive. The procedure was post postponed until after she delivered and interval ultrasounds were obtained throughout her pregnancy to monitor the renal mass. The lesion increased minimally in size. Two months after caesarian section delivery, patient underwent an unremarkable right laparoscopic partial nephrectomy with individual artery and vein vascular occlusion at the level of the renal hilum. A superficial liver laceration caused by the Veress needle was noted at the beginning of the procedure and effectively managed using bipolar cautery. The patient's post-operative course was notable for marked thrombocytosis with her platelet count increasing for her baseline of 300,000 to a zenith of 1.3 million. She was started on aspirin therapy and splenomegaly was noted on abdomen ultrasound. Her platelet count normalized to 334,000. On pathology, the renal mass was classified as a papillary renal neoplasm consistent with a translocation carcinoma. There was no splenic tissue in the specimen. It was noted that the complete lack of staining of any keratins or epithelial membrane antigens was not consistent with a usual renal cell carcinoma and hence the diagnosis of a translocation tumor was made. The patient was lost to follow up presenting 2 years later and a follow-up CT scan noted marked splenic atrophy with several splenules (Figure 3, Figure 4). The patient was completely asymptomatic and at the time received all the post-splenectomy vaccination and her platelet count remained within normal limits.

## Discussion

The spleen is a major lymphopoietic organ and contains approximately 25% of the total lymphoid mass in the body [1]. A major function of the spleen is the removal of particulates and senescent red cells from the blood stream [2]. Post- splenectomy, patients become susceptible to bacterial sepsis due to encapsulated organisms. Patients with acute splenic infarction classically present with left sided upper abdominal pain and tenderness, fever, vomiting, elevated lactate dehydrogenase and leukocytosis. Etiologies include: hypercoagulable states, embolic disease, underlying myeloproliferative neoplasm, hemoglobinopathy, trauma, splenic artery torsion and as complication of infectious mononucleosis. The marked thrombocytosis described in our case study occurred in the immediate post-operative period and suggests that the process started at that time. When splenic infarction is suspected and if the patient has is otherwise asymptomatic, monitoring the coagulation cascade and conservative management are the preferred treatment strategies and surgical management is reserved for life-threatening complications [3].

## Conclusion

Splenic infarction after contralateral right renal surgery is rare and has never been reported. CT imaging is helpful in establishing such a diagnosis and supportive care rather than surgical treatment is preferred.

## Competing interests

The authors declare no competing interest.
